# Retraction: Reduction of neuronal damage and promotion of locomotor recovery after spinal cord injury by early administration of methylprednisolone: possible involvement of autophagy pathway

**DOI:** 10.1039/d2ra90087d

**Published:** 2022-09-20

**Authors:** Yichao Jin, Shaofeng Yang, Xiaohua Zhang

**Affiliations:** Department of Neurosurgery, Shanghai Renji Hospital, Shanghai Jiaotong University, School of Medicine Shanghai 200127 People's Republic of China zhangxh1969@hotmail.com +86-21-58394262 +86-21-34506561

## Abstract

Retraction of ‘Reduction of neuronal damage and promotion of locomotor recovery after spinal cord injury by early administration of methylprednisolone: possible involvement of autophagy pathway’ by Yichao Jin *et al.*, *RSC Adv.*, 2017, **7**, 2979–2991, https://doi.org/10.1039/C6RA25794A.

The Royal Society of Chemistry hereby wholly retracts this *RSC Advances* article due to concerns with the reliability of the data.

The ‘Saline 6 h’ and ‘SCI 24 h’ panels in Fig. 2 are identical, but the images represent different experiments.

The red cells in the ‘Saline 24 h’ panel in Fig. 4 are duplicated in the ‘LC3’ (left), ‘NeuN’ and ‘Merge-1’ panels in Fig. 7, but the images represent different experiments.

The red cells in the ‘SCI 24 h’ panel in Fig. 5 are duplicated in the ‘Beclin-1’ (left), ‘NeuN’ and ‘Merge-1’ panels in Fig. 8, but the images represent different experiments.

The authors have provided raw data for the article. However, the raw data provided for the western blot images was not uncut and unprocessed and therefore could not be used as verifiable raw data to validate the published images. Analysis of the raw data revealed additional bands that were hidden, indicating that the authors have misrepresented the data to select their results.

Given the significance of the concerns about the validity of the data, and the lack of reliable raw data, the findings presented in this paper are not reliable.

The authors were informed but have not responded to any correspondence regarding the retraction.

Signed: Laura Fisher, Executive Editor, *RSC Advances*

Date: 18th August 2022

## Supplementary Material

